# Utilization of five data mining algorithms combined with simplified preprocessing to establish reference intervals of thyroid-related hormones for non-elderly adults

**DOI:** 10.1186/s12874-023-01898-5

**Published:** 2023-05-02

**Authors:** Jian Zhong, Chaochao Ma, Li’an Hou, Yicong Yin, Fang Zhao, Yingying Hu, Ailing Song, Danchen Wang, Lei Li, Xinqi Cheng, Ling Qiu

**Affiliations:** 1 Department of Laboratory Medicine, Peking Union Medical College Hospital, Peking Union Medical College & Chinese Academy of Medical Sciences, Beijing, 100730 China; 2 Department of Laboratory Medicine,, State Key Laboratory of Complex Severe and Rare Diseases, Peking Union Medical College Hospital, Peking Union Medical College & Chinese Academy of Medical Sciences, No. 1 Shuaifu Yuan, Dongcheng District, Beijing, 100730 China

**Keywords:** Algorithms, Data mining, Reference interval, Thyroid-related hormones

## Abstract

**Background:**

Despite the extensive research on data mining algorithms, there is still a lack of a standard protocol to evaluate the performance of the existing algorithms. Therefore, the study aims to provide a novel procedure that combines data mining algorithms and simplified preprocessing to establish reference intervals (RIs), with the performance of five algorithms assessed objectively as well.

**Methods:**

Two data sets were derived from the population undergoing a physical examination. Hoffmann, Bhattacharya, Expectation Maximum (EM), kosmic, and refineR algorithms combined with two-step data preprocessing respectively were implemented in the Test data set to establish RIs for thyroid-related hormones. Algorithm-calculated RIs were compared with the standard RIs calculated from the Reference data set in which reference individuals were selected following strict inclusion and exclusion criteria. Objective assessment of the methods is implemented by the bias ratio (BR) matrix.

**Results:**

RIs of thyroid-related hormones are established. There is a high consistency between TSH RIs established by the EM algorithm and the standard TSH RIs (BR = 0.063), although EM algorithms seems to perform poor on other hormones. RIs calculated by Hoffmann, Bhattacharya, and refineR methods for free and total triiodo-thyronine, free and total thyroxine respectively are close and match the standard RIs.

**Conclusion:**

An effective approach for objectively evaluating the performance of the algorithm based on the BR matrix is established. EM algorithm combined with simplified preprocessing can handle data with significant skewness, but its performance is limited in other scenarios. The other four algorithms perform well for data with Gaussian or near-Gaussian distribution. Using the appropriate algorithm based on the data distribution characteristics is recommended.

**Supplementary Information:**

The online version contains supplementary material available at 10.1186/s12874-023-01898-5.

## Background

Thyroid diseases are prevalent conditions that can cause profound advert consequences, with the global prevalence of clinical hyperthyroidism and hypothyroidism ranging from 0.2 to 1.3% and 0.2 to 5.3%, respectively [1–3]. The relatively high prevalence of the disease implies that regular testing of thyroid-related hormones is essential for early diagnosis and treatment [4, 5]. Given the vital role of reference intervals (RIs) in the correct interpretation of results, it is also necessary for clinical laboratories to establish appropriate RIs to advance clinical practice.

Currently, the direct approach and indirect approach are the main methods used for RI establishment. Traditionally, the direct approach requires a tedious, costly, and time-consuming process to recruit enough presumably healthy individuals. Strict inclusion and exclusion criteria are often required for the definition of so-called healthy individuals. Such strict restrictions often result in inadequate sample sizes available and financial challenges. Thus, it is common for some laboratories to use RIs from other research or manufacturer’s instructions that may not meet the reality of local populations and laboratory conditions [6, 7]. To make up for the aforementioned shortfall in the direct approach, the indirect method may be a preference for clinical laboratories. It utilizes data mining algorithms to analyze the data derived from the routine measurement, known as real-world data (RWD) [8] for RIs establishment. The indirect approach is based on the assumption that the majority of the RWD is derived from non-pathological individuals [9] and the utilization of a robust data mining algorithm can distinguish the distribution of healthy people in the mixed distribution [10, 11]. The alleviation of the difficulty in data collection makes the process of RIs establishment more economical and flexible. RIs generated by the indirect approach is considered applicable to the “intended-to-test” population in the actual clinical setting [10, 12]

Data mining algorithms play an essential role in the implementation of the indirect approach, which helps to make the process cheaper, faster, and more feasible. Hoffmann, Bhattachary, Expectation-Maximum (EM), kosmic, and refineR algorithms are all current mainstream algorithms applied for establishing RIs based on different principles. Despite being proposed early on, Hoffmann [13] and Bhattacharya algorithms [14], are two of the most widely used graph-based methods which can be easily and intuitive understood. The application of these two graphical algorithms is based on the assumption that a large proportion of healthy individuals with Gaussian distribution or near Gaussian distributions exist in the mixed data. Other more recent algorithms, like the EM [15], kosmic [16], and refineR [10] algorithms are based on iteration or parameter searches, and thus may have a differential modeling performance. The EM algorithm is an iteration algorithm with strong operability, which can gain relatively objective and reasonable results by setting “convergence condition”, however, it is difficult to understand the arithmetic principles, producing difficulties in setting parameters. Kosmic and refineR algorithms are recently provided methods based on parametric approach, which can process skewed or non-Gaussian distribution after Box-Cox transformation. In addition to the effect of distribution, the proportion of pathological data may also have an impact on the performance of the algorithm, thus data from the medical examination population is more favored.

To date, it is noted that studies to objectively evaluate the performance of algorithms on dealing with the clinical data are scarce. Previous studies had a complex protocol of data preprocessing combined with a data mining algorithm and habitually judged the plausibility by the comparison with RIs obtained from the direct approach [11, 17–19]. However, this comparison is difficult to be conducted because much time and finanial support may be required. Inspired by the concept of benchmarking in the computer science fields, Tatjana Ammer et al. [20] provided RIbench, a novel benchmarking suite for evaluating the existing indirect methods through simulated test sets. However, as stated by the authors, clinical data are diverse and complex, so the effectiveness of this benchmarking suite in evaluating existing indirect methods for treating authentic clinical data with multimodal pathological distribution is under-research. Besides, heterogeneity in various data preprocessing may also confuse the merits of algorithms. Therefore, the study aims to establish RIs for thyroid-related hormones using five data mining paths based on the indirect approach with simplified data preprocessing for non-elderly adults. The applicability is also appraised by comparing the RIs with those derived from reference individuals after rigorous inclusion and exclusion. Our study is able to provide a methodology reference for the use of an indirect approach to establish RIs.

## Method and materials

### Study design and subjects

**Fig. 1:**
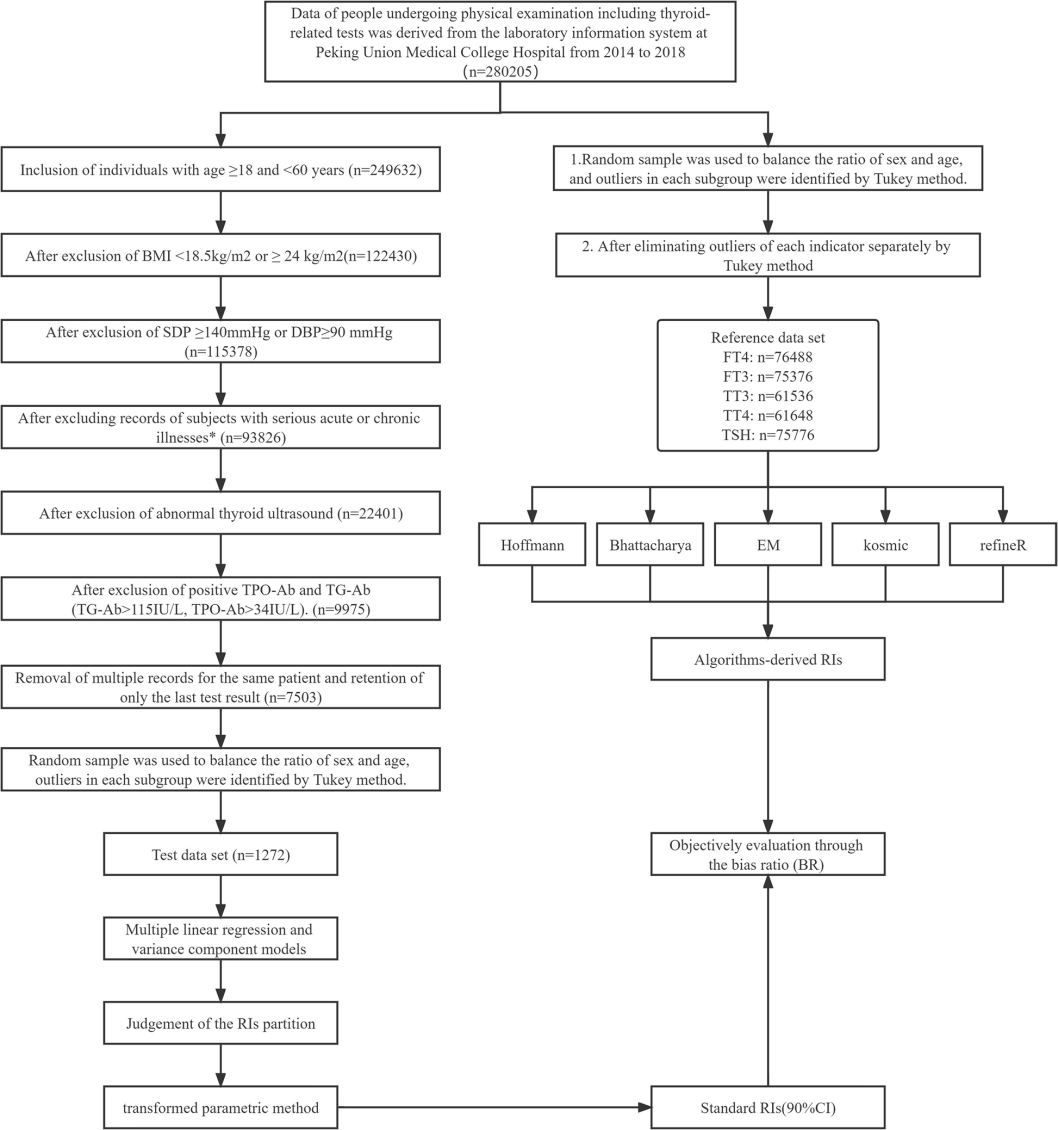
Flowchart of the study design. Detail description of the study design are provided in the Method section. Abbreviation: SBP, systolic blood pressure, DBP, diastolic blood pessure; TPO-Ab, thyroid peroxidase antibody; Tg-Ab, thyroglobulin antibody; FT4, free thyroxine; TT4, total thyroxine; FT3, free triiodothyronine; TT3, total triiodothyronine; Serious acute or chronic illnesses* refer to previous or current serious circulatory, respiratory, urinary, digestive, autoimmune, metabolic, nutritional, hematological, or endocrine diseases, acute or chronic infections, and tumors

Reference data set and Test data set were established in the study. The flowchart was shown in Fig. 1. The inclusion criteria for reference individuals in the Reference data set were listed as follows:

1. Individuals underwent physical examination in the Peking Union Medical College Hospital between January 1, 2014, and December 31, 2018;
2. Age ≥ 18 and < 60 years;

The exclusion criteria for reference individuals were listed as follows:

1. BMI < 18.5 kg/m^2^ or ≥ 24 kg/m^2^;
2. Systolic blood pressure ≥ 140 mmHg or diastolic blood pressure ≥ 90 mmHg;
3. Current or the previous serious circulatory, respiratory, urinary, digestive, autoimmune, metabolic, nutritional, hematological or endocrine diseases, acute or chronic infections, tumors;
4. Abnormal thyroid ultrasound results;
5. Positive TPO-Ab and TG-Ab (TG-Ab > 115 IU/L, TPO-Ab > 34 IU/L).
6. For individuals with repeat test results, the value was retained for the last result.

The sex ratio and age composition of the Reference data set were adjusted by random sampling. Finally, 1272 reference individuals were enrolled in the Reference data set. 

The Test data set was built in a simplified way based on data from the laboratory information system. Results of thyroid-related hormones and other necessary demographic data such as sex and age of people undergoing physical examination from 2014 to 2018 were derived from the laboratory information system at the Peking Union Medical College Hospital, as shown in Fig. 1. To construct the Test data set for calculating the reference interval, only a two-step simplified process was performed without applying the strict inclusion and exclusion criteria described above. The first step is to conduct a random sampling strategy to balance the ratio of sex and age, and the second step is to identify the outliers of variables in each subgroup by the Tukey method. Finally, processed data concerning five hormones, including free thyroxine (FT4), total thyroxine (TT4), free triiodo-thyronine (FT3), total triiodo-thyronine (TT3), and thyroid stimulating hormone (TSH) was obtained for subsequent evaluation.

The transformed parametric method (TP) was used to establish standard RIs for thyroid-related hormones based on the Reference data set, while five data mining algorithms were used for the establishment of RIs based on the Test data set. RIs established by different algorithms were compared with the standard RIs.

### Analytical performance of analytes

The procedure of collecting and detecting fasting blood samples to produce data concerning thyroid-related hormones and antibodies was similar to the previously published study [18]. Fasting blood samples were collected by vacuum into a procoagulant blood collection tube (Vacuette, Greiner Bio-One GmbH, Frickenhausen, Germany). After clotting at room temperature for approximately 30 min, the samples were centrifuged at 2163 g x 10 min to isolate sera. Sera were collected for thyroid-related hormone testing immediately and detection of TSH, FT4, FT3, TT3, and TT4 was performed by an ADVIA Centaur XP chemiluminescence immunoassay analyzer (Siemens Healthineers, Erlangen, Germany). The reagents and calibrators were provided by manufacturers. Instruments remained stable throughout the study period.

### Data collection and quality control

The demographics, clinical laboratory data, and related information for each participant were obtained from the Laboratory Information System and the Hospital physical examination information system. To ensure the stability of the testing results, we maintained the instruments regularly and quality control products were routinely measured to inspect the specimen testing process before each day's work. An internal quality control (QC) data set was used to ensure the correctness and reliability of the results. Moreover, our laboratory has been certified by the International organization for standardization 15,189 (ISO 15189) and the COLLEGE OF AMERICAN PATHOLOGISTS (CAP). Results derived from statistical analysis software or coding running were double-checked to ensure the correctness of the results. Code or R packages in our study have been reported and validated previously [10, 16, 21].

### Data cleaning and statistical analysis

Data storage was carried out by using Excel 2016 (Microsoft, Redmond, WA, USA). Sex was divided into male and female, and age was divided into four groups, including 18–29 years, 30–39 years, 40–49 years, and 50–59 years. We used both R (version 4.0.5) and Medcalc Statistical Software 18.116.6 (Mariakerke, Belgium) to analyze the data.

Box-Cox transformation was implemented in R (version 8.15) with the forecast package before identifying outliers and when establishing RIs to improve the data distribution. Tukey method was used to identify outliers. Furthermore, multiple linear regression and variance component models were used to calculate the standardized regression coefficients and variance of thyroid-related hormones in age and sex respectively. The individual variation was represented as residual standard derivation (SDresidual). To judge the factors influencing the partition of RIs, the standard derivation ratio (SDR) calculated as SDsex /SDresidual and SDage /SDresidual was employed and SDR > 0.4 were set as the cut-off value of partition. RIs and 90% confidence interval (CI) of thyroid-related hormones based on the Reference data set were calculated using a transformed parametric method.

Five data mining algorithms, including Hoffmann, Bhattacharya, Expectation-Maximum (EM), kosmic, and refineR were used to establish RIs of thyroid-related hormones based on the Test data set. These five algorithms were described as follows:

1. Hoffmann algorithm [13]: a classic graph-based algorithm that was developed in 1963 to identify discrepant subsets in a data set with a Gaussian or near-Gaussian distribution. All obtained healthy-related data could be depicted as a scatter plot on the probability paper with the test value as the horizontal coordinate and the cumulative probability or the z-value as the vertical coordinate. This plot was achieved by R code in our study. With the visually judgement of the researcher firstly, the linear region was selected to represent the mainstream distribution of healthy subsets. And the corresponding x-values at 2.5 (z = -1.96) and 97.5 (z = 1.96) on the y-axis were defined as the lower and upper limits of the RI. After the reference intervals were determined by the above user-dependent method, a bootstrap procedure was used to calculate the 90% CIs of the limits which rely on the random sampling of the same database for one hundred replicates. In this process, the linear region used to calculate limits was independently and visually determined in each run.
2. Bhattacharya algorithm [14]: a graphical algorithm similar to the Hoffmann method used for identifying the Gaussian distribution in the mixed data set. The logarithmic transformation of the normal distribution density function leads to the formula, which is $\frac{\mathrm{dln}\left(\mathrm{Y}\left(\mathrm{x}\right)\right)}{\mathrm{dx}}=-\frac{1}{{\sigma }^{2}}\times +\frac{\mu }{{\sigma }^{2}}$. After plotting the corresponding scatter plot by hand or computer programs, the fitted linearity representing the health distribution was visually defined and used for further calculation. We performed visual oversight before using the Bhattcharya analysis and the linear region was selected by visual oversight. The mean and standard deviation of the data distribution can be obtained based on the slope and intercept of the user-defined straight line, thus calculating RI. The method is user-dependent, which requires the user to select the bin size, bin location, and the number of bins for the sake of gaining the best fit line [6]. It can be implemented in R language as Kevin A. Buhr et al. has stated [20]. After the reference intervals were determined by the above user-dependent method, a bootstrap procedure was used to calculated the 90% CIs of the limits which rely on the random sampling of the same database for one hundred replicates. In this process, the linear region used to calculate limits was independently and visually determined in each run.
3. Expectation-Maximum algorithm [15]: an iterative algorithm that consists of two steps in each iteration to determine the best model used to separate the discrepant distributions. The Expectation step determines the parameters of the different models and speculates the probability of each value being assigned to the corresponding model. Maximum step completes the estimation of the model parameters on the basis of the Expectation step. These two steps form a complete iteration. The iteration process will not cease until the pre-defined converge conditions are reached. Once the EM iteration is implemented by using the mixtools package (version1.2.0), the mean and standard deviation of the discrepant distribution can be obtained and used to calculate RIs. Before using EM algorithms, the Box-Cox algorithm was used to improve the normality of data.
4. kosmic algorithm [16]: The approach allows modeling based on the Power Normal distribution, a Gaussian distribution obtained after the Box-Cox transformation of the input physiological data, in which case the effect of the abnormal data is negligible. Furthermore, the estimated distribution can be used for calculating RIs while the minimum Kolmogorov–Smirnov (KS) distance between the estimated normal distribution and a truncated part of the observed distribution is obtained. Tidykosmic package (version 0S.0.0.9000) and kosmic function are used to establish RIs in this part.
5. refineR algorithm[10]: an inverse modeling approach that consists of three steps: region selection of the parameter search and test results, parameter adjustment for model optimization, implement of optimization models to calculate RIs. The refineR package (version 1.0.0) and resRI, getRI function were used in this part.

The RIs of thyroid-related hormones calculated by the transformed parametric method in the Reference data set were defined as the standard RIs in our study, which were compared with those calculated by five data mining algorithms in the Test data set. The bias ratio (BR) matrix was utilized to analyze the discrepancies between the LLs and the ULs among RIs established by different methods. LL and UL in the formula correspond to the lower limit and upper limit of RIs established using the five data mining algorithms in the Test data set, while LL_0_ and UL_0_ in the formula refer to the lower limit and upper limit of the standard RIs. The BR threshold was set to 0.375. Finally, BR values were listed in the BR matrix, which is convenient for a more intuitive comparison.

$$\begin{array}{ccc}{BR}_{LL}=\left|\frac{LL-{LL}_{0}}{{SD}_{RI}}\right|& {BR}_{UL}=\left|\frac{UL-{UL}_{0}}{{SD}_{RI}}\right|& {SD}_{RI}= \frac{{UL}_{0}-{LL}_{0}}{3.92}\end{array}$$

## Results

### The basic information of the Reference and Test data set

The Reference data set and Test data set were derived from the homogeneous data sources in our study and details were demonstrated in Fig. 1. After adjusting the sex and age ratio, both data sets had the same age composition and sex ratio. The sex ratios of the two data sets were both 1:1, and the medians of age were both 40 years. All the reference individuals in the Reference data set were negative for TPO-Ab and TG-Ab (Supplemental Table 1).

### Effect of sex and age on thyroid-associated hormones

The levels of the all five thyroid-related hormones were significantly different in sex, with the male having lower TSH and higher FT3, FT4, TT3, and TT4 compared to the female (*P* ≤ 0.001, Supplemental Fig. 1). Further analysis of the multiple linear regression and the variance component indicated that RIs for FT3 and FT4 should be partitioned by sex as the SDRsex was 0.727 and 0.499, respectively. All the SDRage were less than 0.4, thus RIs for them were not partitioned by age (Supplemental Tables 2 and 3).

### Comparing RIs for thyroid-associated hormones by five algorithms

**Fig. 2:**
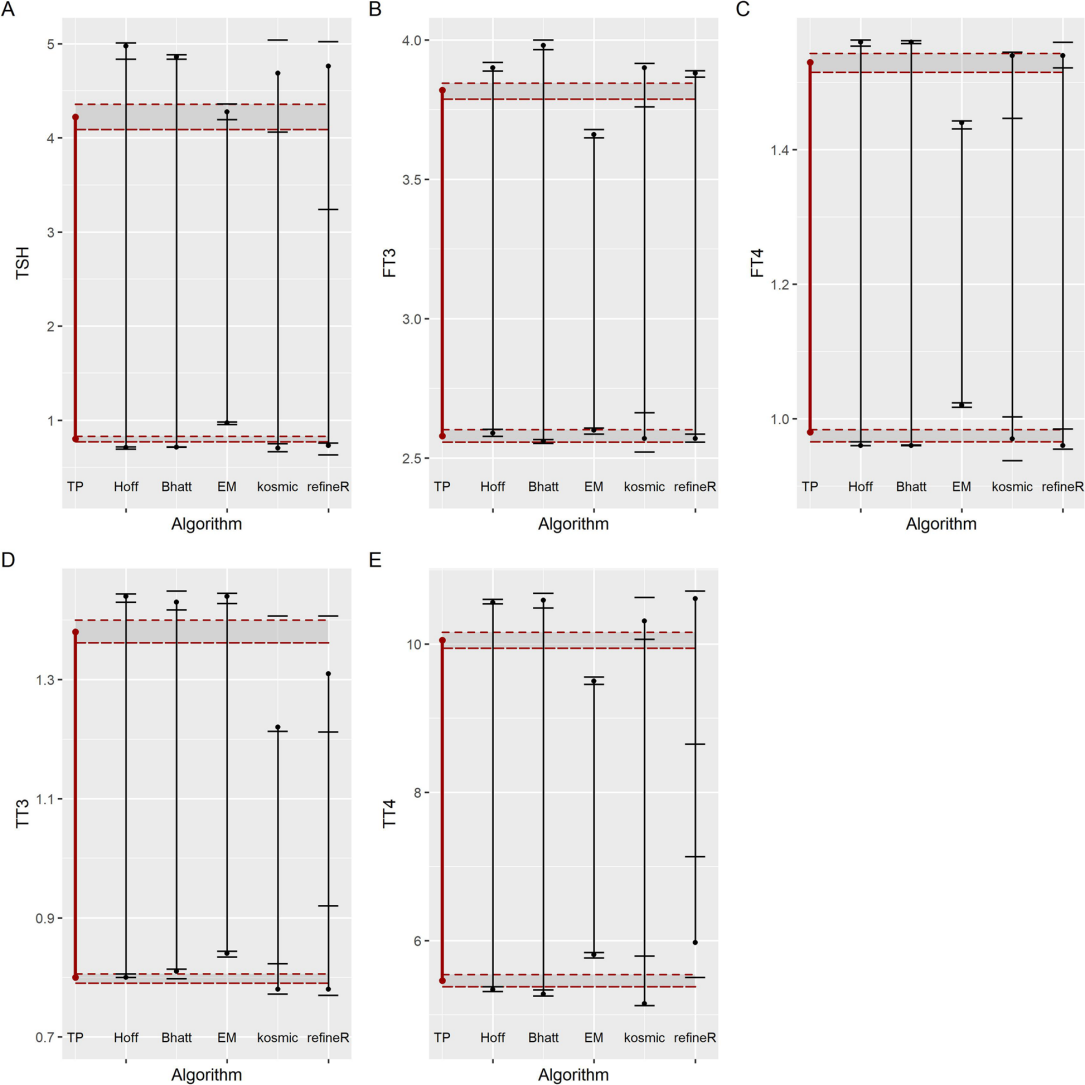
Graphical comparison of algorithm-calculated RIs and standard RIs. **A**-**E** show the algorithm-calculated RIs (and their CIs) for thyroid stimulating hormone(TSH), free triiodothyronine (FT3), free thyroxine (FT4), total triiodothyronine (TT3) and total thyroxine (TT4) in comparison to the standard RIs (and their CIs). The red vertical line indicates the standard RI calculated by transformed parametric method while the black vertical lines stand for the RI calculated by data mining algorithms. The CIs of the standard RIs and algorithm-derived RIs are shown by the horizontal red long dashed and the black short dashed line, respectively

**Fig. 3:**
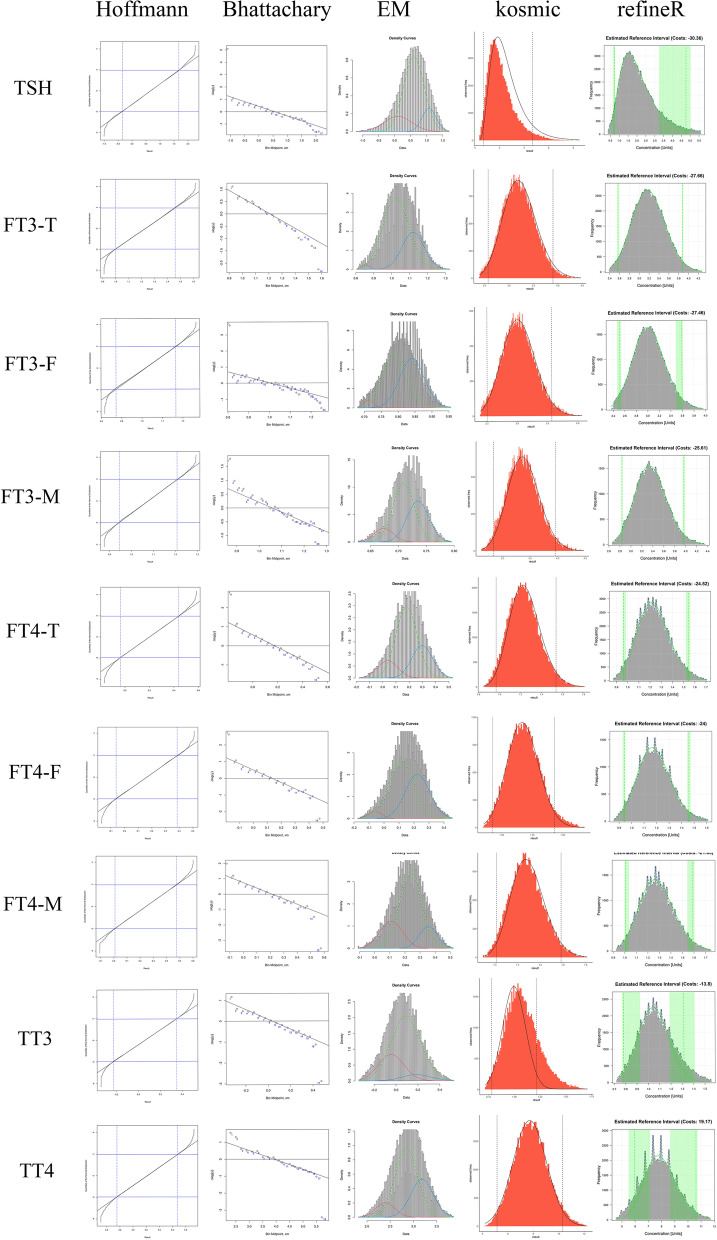
The diagram of algorithms for RIs establishment using the Test data set. Hoffmann and Bhattacharya distinguish the distribution of the healthy individuals from the mixed distribution with the linear region modeling visually. RIs for healthy subgroup are obtained by extending the linear region in these two methods. For Expectation-Maximum algorithm, the diagrams described the distribution of the mixed values after the Box-cox transformation. The green curve denotes the distribution of the healthy population while the blue and red curve represent the distribution of the pathological population. For kosmic, the distribution curve that stands for the estimated distribution is used for establishing RIs. For refineR, the distribution of the healthy population is denoted as green curve, with the green vertical dotted line indicating the limits of the RIs and the shaded vertical areas indicating the 90% confidence interval

The standard RIs for thyroid-associated hormones were obtained from the Reference data set after employing the transformed parametric method, with 90% CI for each limit lower than 0.2 times the width of the RI (Table 1). Hoffmann and Bhattacharya calculated RIs for FT3, FT4, TT3 and TT4 were highly consistent with the standard RIs, while these two algorithms tend to calculate a wider RI for the right-skewed data, TSH. RIs established by the EM algorithm were all narrower than standard RIs, which meant that the EM-derived RIs might have relatively high lower limits (LLs) and/or lower upper limits (ULs) compared to standard RIs. RIs calculated by kosmic and refineR algorithms were close. RIs of FT3 and FT4 calculated by kosmic and refineR were similar to standard RIs, but both algorithms calculated lower LLs and higher ULs for TSH and lower UL for TT3 (Fig. 2). The algorithm models used for RIs establishment are shown schematically in Fig. 3.

**Table 1:** Reference interval established by different methods

		**Reference data set**				**Test data set**							
		**Transformed parametric method**				**Hoffmann**				**Bhattacharya**			
		**LL**	**90%CI**	**UL**	**90%CI**	**LL**	**90%CI**	**UL**	**90%CI**	**LL**	**90%CI**	**UL**	**90%CI**
TSH (μIU/L)	T	0.801	0.7722–0.8305	4.221	4.0900–4.3570	0.709	0.6930–0.7173	4.976	4.8361–5.0115	0.714	0.7120–0.7154	4.861	4.8380–4.8847
FT3 (pg/mL)	T	2.58	2.558–2.602	3.82	3.787–3.845	2.59	2.578–2.604	3.90	3.888–3.919	2.56	2.553–2.567	3.98	3.965–3.999
	F	2.49	2.457–2.519	3.49	3.464–3.517	2.54	2.536–2.581	3.60	3.596–3.608	2.53	2.530–2.532	3.60	3.595–3.600
	M	2.83	2.805–2.855	3.93	3.895–3.976	2.85	2.841–2.868	3.98	3.975–3.988	2.77	2.765–2.770	4.02	3.983–4.077
FT4 (ng/dL)	T	0.98	0.966–0.984	1.53	1.515–1.543	0.96	0.960–0.966	1.56	1.554–1.563	0.96	0.960–0.961	1.56	1.558–1.562
	F	0.95	0.942–0.966	1.42	1.400–1.430	0.94	0.939–0.945	1.48	1.472–1.480	0.94	0.936–0.940	1.48	1.472–1.480
	M	1.01	1.000–1.028	1.58	1.557–1.594	1.01	1.007–1.013	1.60	1.596–1.604	1.01^a^	1.010–1.010	1.60	1.593–1.603
TT3 (ng/mL)	T	0.80	0.790–0.806	1.38	1.362–1.400	0.80	0.800–0.806	1.44	1.430–1.444	0.81	0.798–0.814	1.43	1.417–1.449
TT4 (μg/dL)	T	5.46	5.379–5.539	10.05	9.944–10.158	5.34	5.311–5.379	10.56	10.54–10.60	5.28	5.252–5.335	10.59	10.487–10.683

		**Expectation maximization**				**kosmic**				**refine R**			
		**LL**	**90%CI**	**UL**	**90%CI**	**LL**	**90%CI**	**UL**	**90%CI**	**LL**	**90%CI**	**UL**	**90%CI**
TSH (μIU/L)	T	0.970	0.9540–0.9823	4.276	4.1950–4.3620	0.704	0.6655–0.7515	4.688	4.0629–5.0397	0.732	0.6332–0.7562	4.762	3.2400–5.0228
FT3 (pg/mL)	T	2.60	2.587–2.608	3.66	3.649–3.678	2.57	2.522–2.663	3.90	3.760–3.916	2.57	2.558–2.587	3.88	3.866–3.889
	F	2.54	2.534–2.549	3.32	3.309–3.330	2.50	2.476–2.549	3.57	3.500–3.594	2.52	2.468–2.537	3.59	3.488–3.612
	M	2.94	2.928–2.943	3.73	3.720–3.743	2.84	2.795–2.873	3.96	3.889–3.967	2.84	2.829–2.850	3.97	3.945–3.980
FT4 (ng/dL)	T	1.02	1.017- 1.024	1.44	1.431–1.443	0.97	0.938–1.003	1.54	1.447–1.545	0.96	0.955–0.985	1.54	1.522–1.560
	F	0.95	0.949–0.956	1.36	1.349–1.361	0.93	0.914–0.950	1.43	1.400–1.465	0.94	0.928–0.950	1.46	1.445–1.470
	M	1.10	1.100–1.108	1.52	1.510–1.522	1.01	0.990–1.055	1.58	1.491–1.592	1.01	0.999–1.036	1.59	1.542–1.603
TT3 (ng/mL)	T	0.84	0.834–0.844	1.44	1.428–1.445	0.78	0.772–0.823	1.22	1.213–1.407	0.78	0.770–0.920	1.31	1.212–1.407
TT4 (μg/dL)	T	5.81	5.765–5.838	9.50	9.457–9.553	5.15	5.125–5.791	10.31	10.063–10.626	5.97	5.501–7.131	10.61	8.647–10.715

^a^Before the implementation of the rounding principle, the original result is 1.007(1.006,1.008)

### BR matrix for the comparison of algorithm-calculated RIs and standard RIs

As shown in Table 2, the BR matrix was utilized for the objective comparison of RIs obtained by five indirect methods and standard RIs. For TSH, RIs established by EM algorithms showed the highest consistency with the standard RIs (BR value = 0.063), while the upper limits obtained by the other four algorithms showed pronounced differences.

**Table 2:**
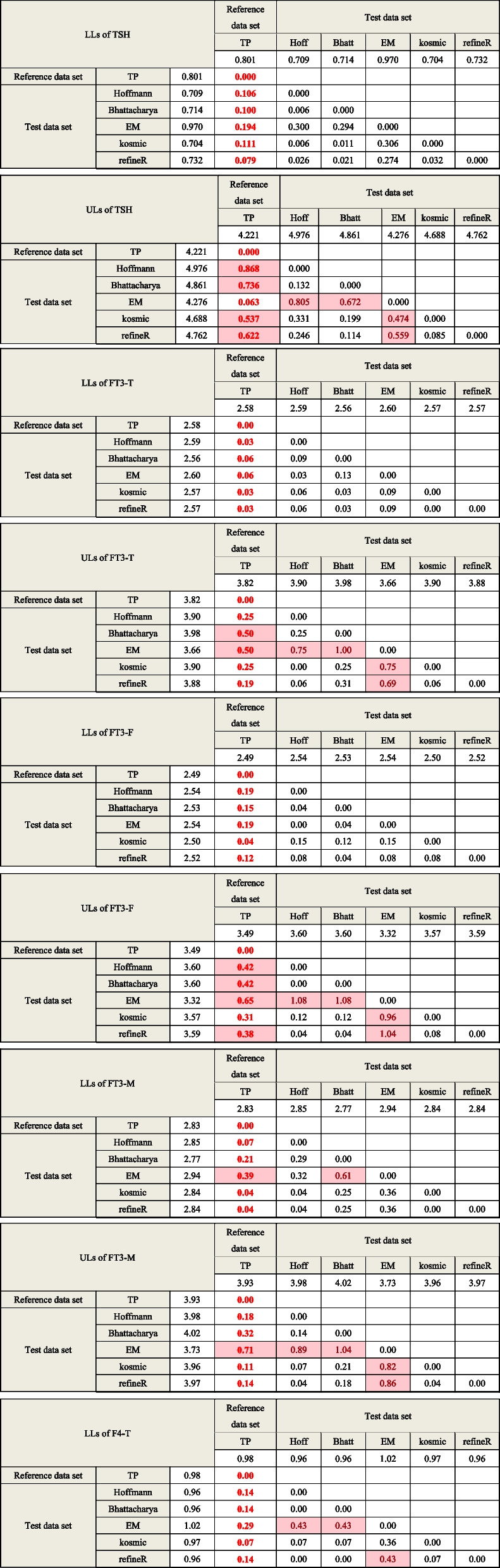

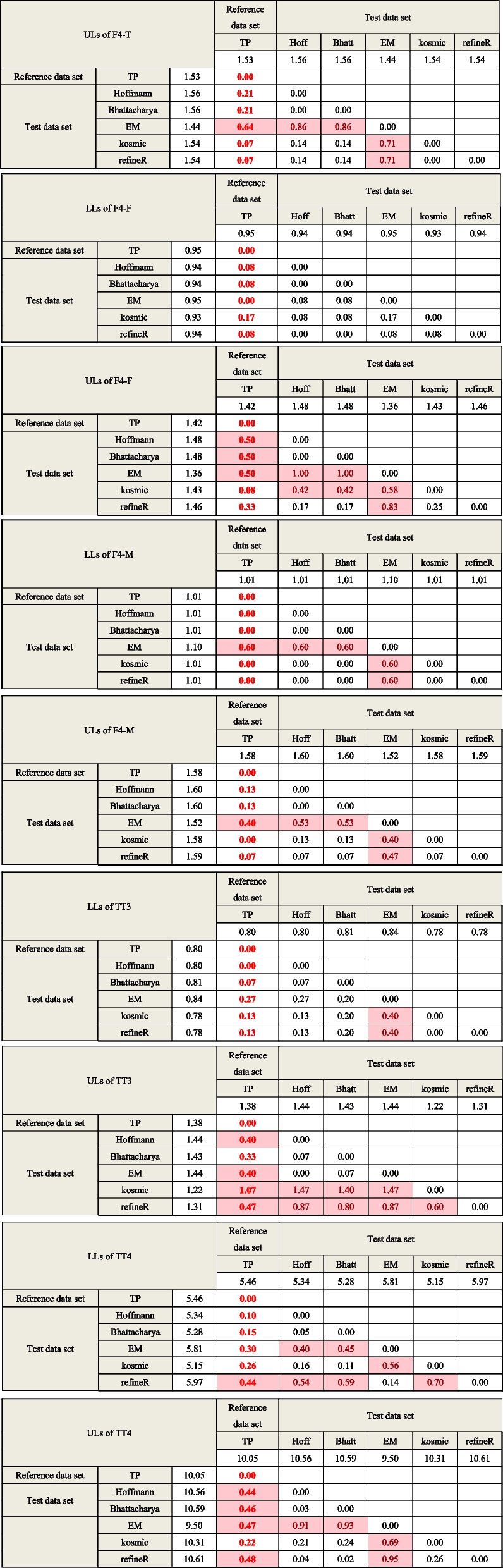
BR matrix for the comparison of different algorithms

Comparison between the algorithm-calculated RIs and standard RIs has been highlighted and bold. BR value exceeds the threshold 0.375 will be marked in red background, indicating that there is a difference between the limits of RIs calculated by two methods from perspective of laboratory medicine

For FT3 and FT4, the RIs calculated by the Hoffmann, Bhattacharya, kosmic, and refineR methods are relatively consistent with the corresponding standard RIs, and the algorithm-calculated RIs for FT3 and FT4 are in high agreement with each other (Table 2). After partitioning by age, the heterogeneity of the RIs for FT3 and FT4 obtained by Hoffmann, Bhattacharya, kosmic, and refineR methods was greater in females than in males, (Fig. 2 and Table 2).

RI for TT4 calculated by kosmic is in line with the standard RI while the other four algorithms-calculated RIs have a slight discrepancy with BR between 0.375–1.0. However, the upper limit of TT3 RI calculated by kosmic is different from standard RI and the calculated BR value is 1.07. Additionally, lower BR values and narrower CIs can be observed when calculating LLs of RIs compared with that of the ULs of RIs.

## Discussion

RIs are important keys for accurate clinical diagnosis and treatment of thyroid diseases. To the best of our knowledge, this is a novel study that simultaneously used five popular algorithms, including Hoffmann, Bhattacharya, Expectation–maximization, kosmic, and refineR algorithms to establish RIs for thyroid-related hormones in non-elderly adults. The combined simplified data preprocessing method and data mining algorithm have high coherence in calculating the RIs of FT3, FT4, and TT4. Among these five algorithms, the EM algorithm combined with the two-step preprocessing shows superior performance in RIs establishment for TSH, a biomarker with the obvious right-skewed distribution, although its performance is limited in dealing with the other thyroid-related hormones.

The development of modern laboratory databases and data mining algorithms implements the indirect approach as more flexible, feasible, and lower cost [8, 22, 23]. Since the fraction and distribution of the pathological distribution can heavily affect the separation capability of the data mining algorithm [10], the physical examination data set with only small amounts of pathological abnormality may be well suited for RIs establishment by the indirect approach. In this study, reference individuals in the Reference data set were selected using strict inclusion and exclusion criteria, while the Test data set used only data concerning thyroid-related hormones after being processed by Box-Cox and Tukey method. It is worth noting that in building Test data set for the implementation of data mining algorithms, the pre-processing steps of data were greatly simplified. Box-Cox and Tukey methods were utilized to exclude outliers in each subgroup rather than in the original aggregate to avoid unnecessary statistical loss. Considering that greater uncertainty and variability may be produced by applying a more pre-analytical process [7, 12], this simplified design allows for a truer reflection of the algorithm’s merits by comparing the consistency between the algorithm-fitted RIs and the standard RIs.

Before the establishment of RIs using the indirect approach, it is vital for us to determine factors influencing the partition of RIs [6]. Because the Reference data set was formed by reference individuals who were selected by strict inclusion and exclusion criteria, we used the Reference data set for analysis of RI partitioning. Multiple linear regression and variance component models [24]were simultaneously used in our study for the discussion of partitioning to ensure the reliability of the results. Finally, we found that partitioning by sex was required for FT3 and FT4 while partitioning by age was unnecessary. To provide more valuable information about the partitioning and establishment of RIs for clinical laboratories, total and sex-specific RIs for FT3 and FT4 were all established and compared in these two data sets.

The transformed parametric method (using log transformation) was utilized for calculating the standard RIs, although there were other alternatives for establishing RIs, such as the non-parametric, and robust methods [25]. This is because robust method is usually used for small sample size calculation and the RIs for TSH can be stable only when the sample size is greater than 850 for non-parametric method [26]. Our previous studies also found that RIs for TSH established by the transformed parametric methods has the smallest variation [27]. Thus, the RI calculated with a suitable data set and a stable method can be used as a yardstick to evaluate the performance of existing algorithms. In our study, it was found that Hoffmann and Bhattacharya calculated negative lower limits of RIs for TSH, which indicated their poor performance in processing data with skewed distribution [7, 21]. This finding echoed the research of Daniel T. Holmes [21]. However, when using Box-Cox transformation algorithms before applying Hoffmann and Bhattacharya algorithms, these two algorithms can obtain reasonable results. In addition, Hoffmann and Bhattacharya algorithms are graphical methods with a subjective selection of the range of linearity, which diminish the possibility of computing RIs automatically. In contrast, the EM algorithm based on the principle of unsupervised clustering could distinguish even very close distribution when the samples were large enough. This algorithm could be easily implemented under the circumstance of R language although the determination of parameters is vital [15]. We found that the EM algorithm combined with the two-step preprocessing performed well in calculating RIs for TSH. Moreover, RIs obtained by kosmic and refineR algorithms for FT3, FT4 were close to standard RIs. It appears that the kosmic algorithm has a limited ability to establish RIs of TT3, as reflected by the significant difference between its fitted TT3 RI and the standard TT3 RI. Combined with simplified two-step data processing, refineR shows stable and relatively consistent performance in calculating RIs for data with Gaussian or moderate-skewed distribution. Moreover, lower BR values and narrower CIs are obtained when calculating LLs of RIs compared with that of the ULs of RIs, indicating that data-mining algorithms combined with simplified data pre-processing methods have an excellent performance in calculating LLs when handling data with near-Gaussian or right-skewed distributions. Furthermore, the heterogeneity of the RIs for FT3 and FT4 obtained by Hoffmann, Bhattacharya, kosmic, and refineR methods was greater in females than in males, which might be explained by the more right-skewed distribution of the FT3 and FT4 for females than males. We infer from these results that data distribution characteristics have an impact on the consistency and accuracy of algorithms. Further studies on the topics of how data distribution characteristics affect the consistency and accuracy of the algorithm and how to apply the algorithm appropriately according to the data distribution characteristics are therefore suggested.

Undoubtedly, there are both advantages and disadvantages to our research. The advantages are listed as follows: 1) a process combining simplified data preprocessing with algorithms is proposed in our study, which can provide a methodological basis for the implementation of indirect methods in clinical laboratories. 2) five algorithms are used respectively to establish RIs for thyroid-related hormones in non-elderly people, and the applicability of the five algorithms is objectively assessed. Performance differences in algorithms suggested that more attention should be paid to the characteristic of data distribution when selecting algorithms. The limitation of this study is that older people were not included in the study. This is because those previous studies have found different levels of thyroid-related hormones between elderly and non-elderly people [28–30] and one study suggest that indirect comparisons need to be made separately in subgroups [31]. Therefore, we just included non-elderly people in this study and analysis of data from older people has been reported in the other research [32].

## Conclusion

Combined with a simplified pre-analysis process, five data mining algorithms are feasible to establish RIs for thyroid-related hormones for non-elderly people. The EM algorithm only has an excellent performance in handling data with significantly skewed distribution such as TSH, while its performance on other hormones is limited. Hoffmann, Bhattacharya, kosmic, and refineR perform well for thyroid-related hormones other than TSH. The differences in RI established by the various algorithms suggest that more attention should be paid to the distributional characteristics of the data when choosing an indirect method.

## Supplementary Information

**Additional file 1:** **Supplemental Table 1.** Basic characteristics of the two datasets. **Supplemental Table 2.** Results of multiple linear regression. **Supplemental Table 3.** Analysis of variance component and SDR. **Supplemental Figure 1.** Distribution of the thyroid-related hormones in the Reference data set. 

## Data Availability

All results presented in this study are objectively shown in this article and/or its Additional file.

## Abbreviations

TSH: Thyroid stimulating hormone
RIs: Reference intervals
EM: Expectation-Maximum algorithm
TP: Transformed parametric method
CI: Confidence interval
SBP: Systolic blood pressure
DBP: Diastolic blood pessure
TPO-Ab: Thyroid peroxidase antibody
Tg-Ab: Thyroglobulin antibody
FT4: Free thyroxine
TT4: Total thyroxine
FT3: Free triiodothyronine
TT3: Total triiodothyronine
