# Supplementary material for: Utilization of five data mining algorithms combined with simplified preprocessing to establish reference intervals of thyroid-related hormones for non-elderly adults
Source: BMC Med Res Methodol. 2023 May 2;23:108. doi: 10.1186/s12874-023-01898-5 (PMC10152698; doi:10.1186/s12874-023-01898-5)
Supplement: Supplementary file 1 — Additional file 1: Supplemental Table 1. Basic characteristics of the two datasets. Supplemental Table 2. Results of multiple linear regression. Supplemental Table 3. Analysis of variance component and SDR. Supplemental Figure 1. Distribution of the thyroid-related hormones in the Reference data set. [file 12874_2023_1898_MOESM1_ESM.docx]

**Supplemental Table 1 Basic characteristics of the two datasets**

| Index | Reference data set | Test data set |
| --- | --- | --- |
| Age (year) | 40 (30,49) | 40 (30,49) |
| Sex ration (Female: Male) | 1:1 | 1:1 |
| BMI (kg/m^2^) | 21.88 (20.41,22.99) | / |
| SBP (mmHg) | 112 (104,121) | / |
| DBP (mmHg) | 70 (64,76) | / |
| TPO-Ab (IU/L) | 12.10 (10.02,14.89) | / |
| TG-Ab (IU/L) | 10.78 (10.00,13.47) | / |

**Supplemental Table 2 Results of** **multiple linear regression**

| **Index** | **Sex** | | **Age** | | | | | |
| --- | --- | --- | --- | --- | --- | --- | --- | --- |
|  | **β** | **P** | **A1** | | **A2** | | **A3** | |
|  |  |  | **β** | **P** | **β** | **P** | **β** | **P** |
| **TSH** | -0.110 | <0.001 | -0.006 | 0.867 | 0.036 | 0.299 | -0.011 | 0.755 |
| **FT3** | 0.522 | <0.001 | -0.044 | 0.129 | -0.142 | <0.001 | -0.146 | <0.001 |
| **FT4** | 0.372 | <0.001 | -0.108 | 0.001 | -0.179 | <0.001 | -0.207 | <0.001 |
| **TT3** | 0.189 | <0.001 | 0.001 | 0.974 | -0.032 | 0.344 | 0.065 | 0.055 |
| **TT4** | 0.095 | 0.001 | -0.042 | 0.215 | -0.076 | 0.025 | 0.006 | 0.864 |

β, Standardized regression coefficient; Female is the reference for sex groups. A1, A2, and A3 are the dummy variable of Age, 18~29 years is the reference level, A1 stands for 30~39 years relative to 18~29 years, and A2 stands for 40~49 years relative to 18~29 years, A3 stands for age 50 ~59 relative to 18~29 years.

**Supplemental Table 3 Analysis of variance component and SDR**

| **Index** | **(UL-LL)/3.92** | **Sex** | | **Age** | |
| --- | --- | --- | --- | --- | --- |
|  |  | **SD** | **SDR** | **SD** | **SDR** |
| **TSH** | 0.872 | 0.134 | 0.154 | 0.000 | 0.000 |
| **FT3** | 0.32 | 0.23 | 0.727 | 0.05 | 0.158 |
| **FT4** | 0.14 | 0.07 | 0.499 | 0.03 | 0.214 |
| **TT3** | 0.15 | 0.04 | 0.270 | 0.01 | 0.068 |
| **TT4** | 1.17 | 0.14 | 0.120 | 0.12 | 0.102 |

SDresidual represents the individual variance and the standard derivation ratio (SDR) is calculated as SDsex /SDresidual and SDsex/SDresidual ). SDR>0.4 are set as the cut-off value of partition, thus the only the RIs for FT3 and FT4 should be partitioned by sex. Age should not be used as a basis for partitioning, as all SDRage is less than 0.4.

**Supplemental Figure 1. Distribution of the thyroid-related hormones in the Reference data set**

**
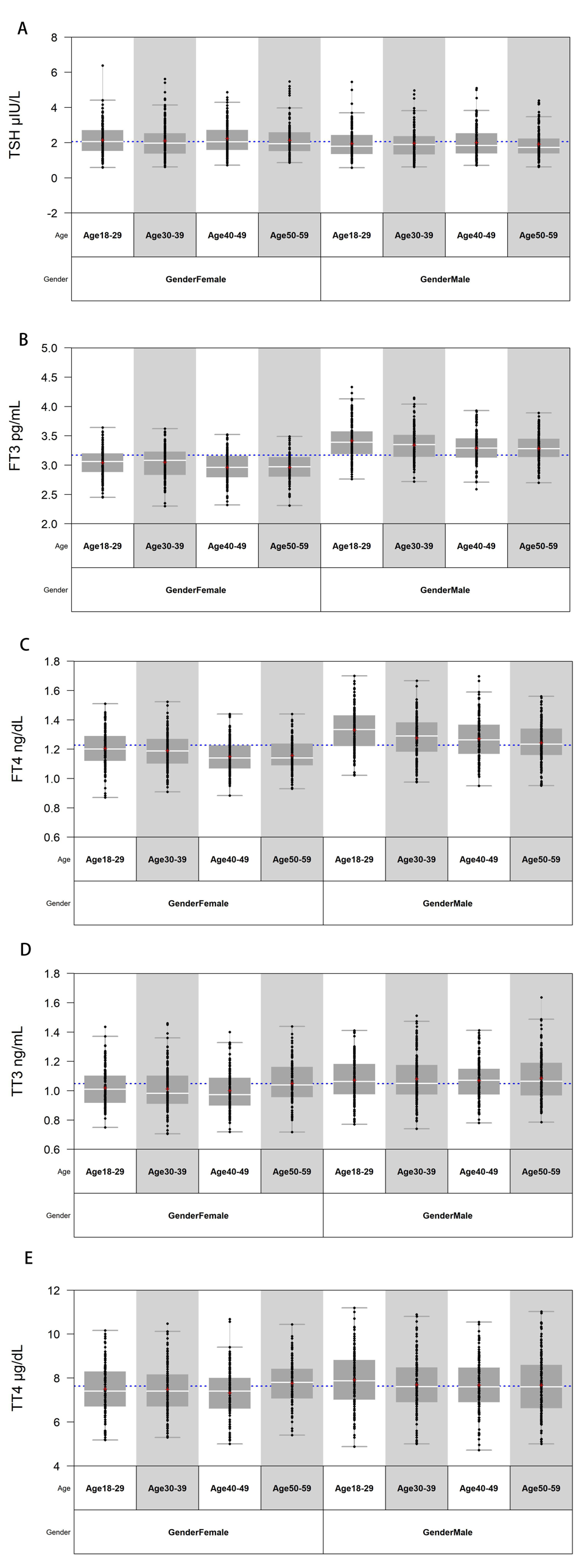
**
